# Short Tandem Repeat Expansions and RNA-Mediated Pathogenesis in Myotonic Dystrophy

**DOI:** 10.3390/ijms20133365

**Published:** 2019-07-09

**Authors:** Łukasz J. Sznajder, Maurice S. Swanson

**Affiliations:** Department of Molecular Genetics and Microbiology, Center for NeuroGenetics and the Genetics Institute, University of Florida, College of Medicine, Gainesville, FL 32610, USA

**Keywords:** myotonic dystrophy, ALS/FTD, microsatellite expansion, STR, alternative splicing, MBNL, RBFOX, CELF, phase separation, foci

## Abstract

Short tandem repeat (STR) or microsatellite, expansions underlie more than 50 hereditary neurological, neuromuscular and other diseases, including myotonic dystrophy types 1 (DM1) and 2 (DM2). Current disease models for DM1 and DM2 propose a common pathomechanism, whereby the transcription of mutant *DMPK* (DM1) and *CNBP* (DM2) genes results in the synthesis of CUG and CCUG repeat expansion (CUG^exp^, CCUG^exp^) RNAs, respectively. These CUG^exp^ and CCUG^exp^ RNAs are toxic since they promote the assembly of ribonucleoprotein (RNP) complexes or RNA foci, leading to sequestration of Muscleblind-like (MBNL) proteins in the nucleus and global dysregulation of the processing, localization and stability of MBNL target RNAs. STR expansion RNAs also form phase-separated gel-like droplets both in vitro and in transiently transfected cells, implicating RNA-RNA multivalent interactions as drivers of RNA foci formation. Importantly, the nucleation and growth of these nuclear foci and transcript misprocessing are reversible processes and thus amenable to therapeutic intervention. In this review, we provide an overview of potential DM1 and DM2 pathomechanisms, followed by a discussion of MBNL functions in RNA processing and how multivalent interactions between expanded STR RNAs and RNA-binding proteins (RBPs) promote RNA foci assembly.

## 1. Introduction

**Figure 1:**
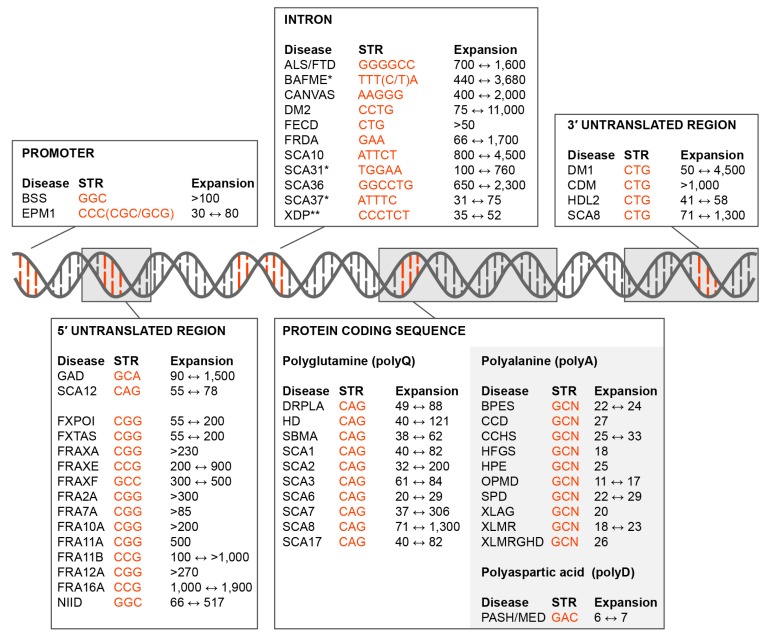
Short tandem repeat (STR) expansion disorders. Diseases caused by STR (orange font) expansions in the promoter, 5’ untranslated region (5’UTR), introns, coding region and 3’ untranslated region (3’UTR) are shown together with the disease acronym and pathogenic expansion range (black font). Some of these mutations are not classical expansions but are insertions due to replication/recombination/duplication (Intron, SCA31, SCA37, BAFME, *; Protein Coding Sequence, Polyalanine and Polyaspartic acid, light grey box) or retrotransposon (Intron, XDP, **) events. Disease-associated STR locations include the: • Promoter. Baratela-Scott Syndrome (BSS) linked to *XYLT1* gene [6], Progressive Myoclonus Epilepsy (EPM1)–*CSTB* [7]; • 5’UTR. Glutaminase Deficiency (GAD)–*GLS* [8]; Spinocerebellar Ataxia (SCA) Type 12 (SCA12)–*PPP2R2B* [9]; Fragile X-Associated Primary Ovarian Insufficiency (FXPOI), Fragile X-Associated Tremor/Ataxia Syndrome (FXTAS) and Fragile XA Syndrome (FRAXA or FXS)–*FMR1* [10,11]; Fragile XE Syndrome (FRAXE)–*AFF2* [12]; Fragile XF Syndrome (FRAXF)–*TMEM185A* [13]; Folate-sensitive fragile sites (FSFS) FRA2A–*AFF3* [14]; FSFS FRA7A–*ZNF713* [15]; FSFS FRA10A–*FRA10AC1* [16]; FSFS FRA11A–*C11orf80* [17]; SFSF FRA11B–*CBL2* [18]; FSFS FRA12A–*DIP2B* [19]; SFSF FRA16A–*LOC109617027* [20]; Neuronal Intranuclear Inclusion Disease (NIID)–*NOTCH2NLC* [21]; • Intron. Amyotrophic Lateral Sclerosis and Frontotemporal Dementia (ALS/FTD)–*C9orf72* [22,23]; Benign Adult Familial Myoclonic Epilepsy (BAFME)–*SAMD12, TNRC6A* and *RAPGEF2* [24]; Cerebellar Ataxia, Neuropathy, Vestibular Areflexia Syndrome (CANVAS)–*RFC1* [25]; Myotonic Dystrophy Type 2 (DM2)–*CNBP* [26]; Fuchs Endothelial Corneal Dystrophy (FECD)–*TCF4* [27]; Friedrich’s Ataxia (FRDA)–*FXN* [28]; SCA type 10 (SCA10)–*ATXN10* [29]; SCA type 31 (SCA31)–*BEAN1/TK2* [30]; SCA36–*NOP56* [31]; SCA37–*DAB1* [32]; X-Linked Dystonia-Parkinsonism (XDP)–*TAF1* [33]. • Coding region (polyglutamine). Dentatorubral-Pallidoluysian Atrophy (DRPLA)–*ATN1* [34]; Huntington Disease (HD)–*HTT* [35]; Spinal and Bulbar Muscular Atrophy (SBMA)–*AR* [36]; SCA type 1 (SCA1)–*ATXN1* [37], SCA type 2 (SCA2)–*ATXN2* [38]; SCA type 3 (SCA3)–*ATXN3* [39]; SCA type 6 (SCA6)–*CACNA1A* [40]; SCA type 7 (SCA7)–*ATXN7* [41]; SCA type 8 (SCA8)–*ATXN8* [42]; SCA type 17 (SCA17)–*TBP* [43]; • Coding region (polyalanine). Blepharophimosis Syndrome (BPES)–*FOXL2* [44]; Cleidocranial Dysplasia (CCD)–*RUNX2* [45]; Congenital Central Hypoventilation Syndrome (CCHS)–*PHOX2B* [46]; Hand-Foot-Genital Syndrome (HFGS)–*HOXA13* [47]; Holoprosencephaly (HPE)–*ZIC2* [48]; Oculopharyngeal Muscular Dystrophy (OPMD)–*PABPN1* [49]; Synpolydactyly Syndrome (SPD)–*HOXD3* [50]; X-linked Mental Retardation and Abnormal Genitalia (XLAG) and X-linked Mental Retardation (XLMR)–*ARX* [51,52]; XLMR and Growth Hormone Deficit (XLMRGHD)–*SOX3* [53]; • Coding region (polyaspartic acid). Pseudoachondroplasia and Multiple Epiphyseal Dysplasia (PSACH/MED)–*COMP* [54]. • 3’UTR. Myotonic Dystrophy Type 1 (DM1) and Congenital Myotonic Dystrophy (CDM)–*DMPK* [55]; Huntington Disease-Like 2 (HDL2)–*JPH3* [56]; SCA8–*ATXN8OS* [42].

Repetitive DNA sequence elements compose 50–70% of the human genome [1,2]. These repetitive sequences include interspersed transposable elements (DNA transposons, RNA retrotransposons) and tandem repeats, including telomeric and centromeric repeats as well as micro-, mini- and mega-, satellites. Microsatellites or short tandem repeats (STRs) composed of 2–10 base pairs (bp), are highly polymorphic in the human population [3]. Their propensity to form unusual quadruplex-like, slipped-stranded structures and imperfect hairpins results in an elevated level of DNA replication and repair errors that can lead to STR contractions or expansions [4,5]. While these repeat expansion length variations may serve to fine-tune regulatory activities of the STR host gene, more than 50 hereditary neurological, neuromuscular and other diseases are associated with expansions in coding and noncoding regions as well as in promoters (Figure 1). STR expansion diseases are caused by a variety of AT- and GC-rich tandem repeat sequences. While GC-rich trinucleotide expansions predominate in exonic regions, intronic repeats are composed of 3-6 nucleotide tandems and vary considerably in GC content.

Although DNA repeat expansions are the primary cause of the associated disorder, the downstream pathomechanisms underlying development of the disease phenotype have remained unclear for the majority of these diseases. Possible STR expansion disease mechanisms include host gene haploinsufficiency, host gene transcript misprocessing [57,58], bidirectional gene transcription [59], repeat-mediated RBP sequestration [60], canonical translation of toxic polyglutamine and polyalanine proteins [61,62] and non-canonical repeat-associated non-AUG (RAN) translation [63]. Of course, these pathomolecular events may and often do, co-occur in an expansion disease. For example, in *C9orf72* amyotrophic lateral sclerosis and frontotemporal dementia (C9-ALS/FTD), the *C9orf72* intron 1 GGGGCC expansion (GGGGCC^exp^) results in alternative first exon selection, intron 1 retention, altered sense and antisense transcription, sequestration of multiple repeat-binding proteins (e.g., HNRNPH1, RanGAP1) and RAN translation of dipeptide repeats (DPRs) [64].

**Figure 2:**
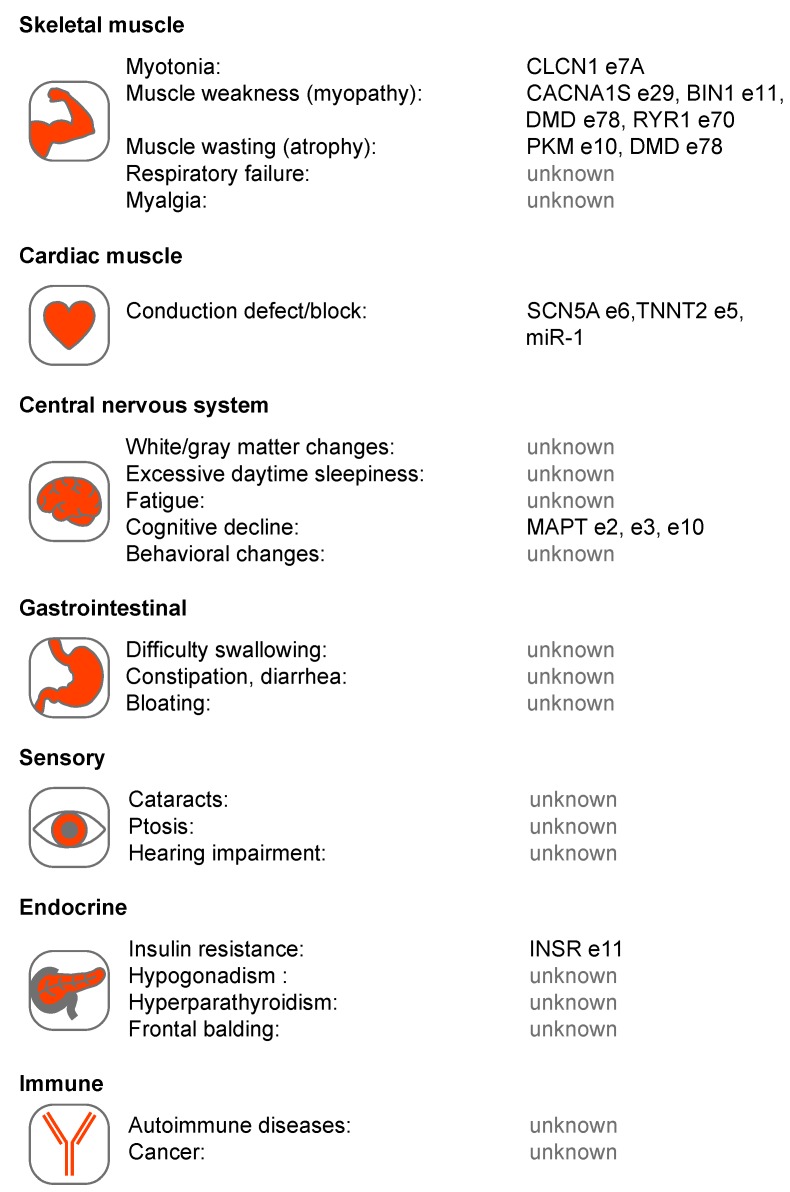
Multi-system involvement in Myotonic Dystrophy types 1 and 2 (DM1 and DM2). Several tissue systems are shown with associated phenotypes together with proposed RNA mis-processing events. These events include: chloride voltage-gated channel 1 (CLCN1) exon (e)7A [81,82,83], calcium voltage-gated channel subunit alpha1 S (CACNA1S) e29 [84], bridging integrator 1 (BIN1) e11 [85], dystrophin (DMD) e78 [86], ryanodine receptor 1 (RYR1) e70 [87], pyruvate kinase isozymes M1/M2 (PKM1/M2) e10 [88], sodium voltage-gated channel alpha subunit 5 (SCN5A) e6 [89,90], troponin T2, cardiac type (TNNT2) e5 [91], microtubule associated protein tau (MAPT) e2, e3, e10 [92,93], insulin receptor (INSR) e11 [94,95] and microRNA-1 (miR-1) [96].

In this review, we focus on two related multisystemic disorders, myotonic dystrophy (DM) type 1 (DM1) and type 2 (DM2), which have served as a paradigm of RNA-mediated diseases. Although DM1 is caused by a *DMPK* 3’ untranslated region (3’UTR) CTG^exp^ and DM2 by an intronic CCTG^exp^ in *CNBP,* they share a number of pathological features including skeletal muscle myotonia and weakness/wasting, heart conduction block, unusual dust-like ocular cataracts and cognitive dysfunction (Figure 2). Below, we evaluate different disease mechanisms for DM1 and DM2 in the context of other microsatellite expansion disorders followed by a more detailed analysis of the pathogenic roles of toxic CUG^exp^ and CCUG^exp^ in RNA processing.

## 2. Is Myotonic Dystrophy Caused by Haploinsufficiency?

Loss-of-function mechanisms are well documented in Friedreich’s ataxia (FRDA) and a number of folate-sensitive fragile sites, including Fragile XA syndrome (FRAXA) where microsatellite expansions induce epigenetic changes that result in transcriptional repression (Figure 1) [65,66]. However, several observations argue against a haploinsufficiency or host gene loss-of-function, model for either DM1 or DM2. First, neither *DMPK* nor *CNBP* coding region mutations have been reported to cause DM. Second, both DM1 and DM2 are classified as myotonic dystrophies but DM1 is caused by *DMPK* CTG^exp^ and DM2 by *CNBP* CCTG^exp^, mutations and these two genes are located on different chromosomes and encode proteins with very different functions. DMPK is a serine/threonine protein kinase while CNBP is a CCHC-type zinc finger (ZnF) protein. *DMPK* is expressed as six major isoforms and is a member of the AGC kinase family [67,68] while CNBP has been implicated in both transcriptional and post-transcriptional regulation and a recent study demonstrated that this ZnF protein binds to G-rich RNA elements to block G-quadruplex structures and enhance translation [69]. Third, the *DMPK* CTG^exp^ mutation results in the retention of DMPK mRNA in the nucleus and depletion of DMPK protein but neither *Dmpk* heterozygous nor homozygous knockout mice recapitulate the major pathological features of DM1 [70,71,72].

For DM2, the CCTG^exp^ mutation does not inhibit *CNBP* transcription [73]. The CCTG^exp^ is located in an intron so splicing should allow nucleocytoplasmic export of CNBP mRNA. Although CNBP intron 1 retention occurs in DM2 this partially spliced mRNA is exported from the nucleus [58]. The effect of the CCTG^exp^ mutation on CNBP protein expression in DM2 is controversial with either no effect [74,75,76] or decreases in CNBP protein levels reported [77,78,79]. Interestingly, CNBP re-localizes from the cytoplasm to the sarcolemma where it interacts with α-dystroglycan in DM2 muscle biopsies, although the molecular basis for CNBP re-localization is unclear since mutant and wild-type *CNBP* alleles produce the same protein [80]. Nevertheless, the discrepancy between CNBP protein levels in muscle biopsies may reflect differential solubility of membrane-associated fractions.

In an effort to model DM2, several *Cnbp* mouse knockout models have been generated that vary in phenotype, possibly due to strain background, which are characterized by either embryonic lethality or sarcomere disorganization and muscle atrophy, in homozygous knockouts while *Cnbp*^+/−^ heterozygous knockouts develop later-onset muscle weakness/wasting [80,97,98]. Thus, *Cnbp* knockouts reproduce skeletal muscle features common to DM1 and DM2 although CNBP levels are not compromised in DM1. CNBP downregulation has also been noted in a zebrafish model of Treacher Collins syndrome, a craniofacial disorder caused by mutations in *TCOF1* that encodes the treacle protein involved in rDNA transcription [99]. Interestingly, forebrain truncation and craniofacial defects have also been observed in *Cnbp* knockout mice [97]. Cumulatively, these findings argue that *DMPK* haploinsufficiency is not a major DM1 disease factor but a potential role for *CNBP* loss-of-function in DM2 requires further study.

## 3. DM1 and DM2 Are RNA-mediated Disorders

In contrast to the dysregulation of host gene expression, transcription of microsatellite expansion mutations independent of the host gene context results in the synthesis of toxic STR RNAs. Thornton and colleagues provided evidence for CUG^exp^ toxicity by the generation and characterization of *HSA*^LR^ transgenic mice, in which a CTG^~250^ mutation was inserted into the 3’UTR of a human skeleton actin (*HSA*) transgene [100]. *HS*A^LR^ mice develop several manifestations of DM1 muscle, including myotonia, with pathological severity dependent on transgene expression level.

Both the RNA sequences and structures of STR expansions have been implicated as pathogenic factors and AU/GC composition influences the propensity of these expansions to form higher order RNA structures. In silico prediction of secondary structures formed by expanded microsatellites indicates that the majority of AU-rich repeats are primarily single-stranded compared to those formed by GC-rich STRs [58,101,102]. For instance, DM1 CUG and DM2 CCUG repeats form stable imperfect hairpins with one or two unpaired nucleotides while the C9-ALS/FTD GGGGCC repeats form G-quadruplex structures [103,104,105]. In addition, inherited mutation length increases during an affected individual’s lifespan, particularly in post-mitotic cells and results in different cells expressing varying repeat lengths [106,107,108,109]. Due to this somatic mosaicism, *DMPK* mutant allele repeat expansions are readily detectable in blood cells but expansions in skeletal muscles may be much larger and reach thousands of CTG repeats with variable repeat lengths in different myonuclei [110,111]. Current evidence indicates somatic expansion is one of the triggers of pathological onset, however in some tissues there is no clear correlation between expansion size and disease severity [112]. Moreover, exceptionally large microsatellite expansions in noncoding regions are not always associated with greater disease severity. For instance, DM2 is caused by up to 11,000 CCTG repeats in *CNBP* intron 1 but this disease is generally recognized as a less severe type of myotonic dystrophy distinguished by relatively late-onset and lack of a congenital form [113].

**Figure 3:**
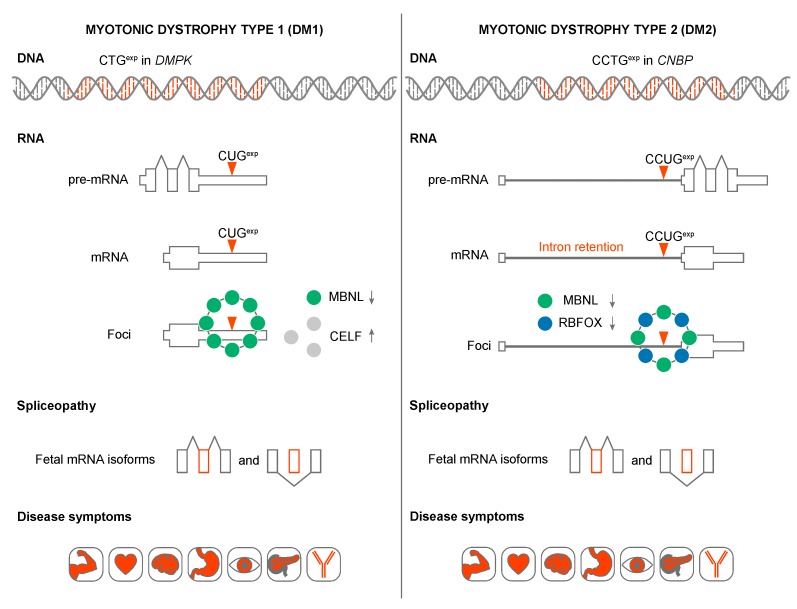
Models of DM1 and DM2 disease mechanisms. CTG^exp^ and CCTG^exp^ in the *DMPK* and *CNBP* genes produce pre-mRNA transcripts containing expanded CUG and CCUG repeats. DMPK pre-mRNA is correctly spliced whereas CCUG^exp^ triggers CNBP intron 1 retention. mRNAs with C(C)UG^exp^ sequester Muscleblind-like (MBNL) and RBFOX (in DM2) alternative splicing factors. In addition, CUG^exp^ increase CUGBP Elav-Like Family Member 1 (CELF1) splicing factor stability through protein kinase C (PKC)-mediated hyperphosphorylation. All these changes in the bioavailability of splicing factors cause an imbalance in alternative splicing and enhanced fetal mRNA isoform production in adult tissues. As a result, inappropriate protein expression patterns lead to a variety of DM symptoms.

Why are STR expansion RNAs toxic? In DM1 and DM2, CUG^exp^ and CCUG^exp^ RNAs adversely affect the activities of several developmentally regulated RNA splicing factors in human tissues, cells and animal models (Figure 3) [114,115]. For example, in DM1 skeletal muscle and heart, expression of CUG^exp^ RNA leads to protein kinase C (PKC)-mediated CELF1 hyperphosphorylation [116,117]. Since CELF1 promotes fetal alternative splicing patterns, CUG^exp^ expression and CELF1 overexpression plays a role in the reversion to fetal splicing patterns in adult tissues [118,119]. These mis-splicing events have been linked to specific pathophysiological outcomes including myotonia, muscle weakness/wasting, heart conduction block and insulin resistance. It is unclear if CELF1 is upregulated in DM2 [79,117,120].

## 4. MBNL Sequestration and Loss-of-function in DM1 and DM2

**Figure 4:**
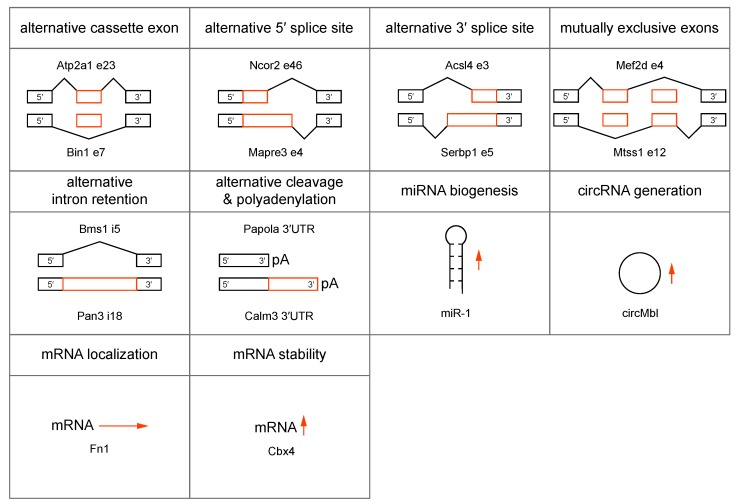
MBNL functions in RNA biogenesis, localization and stability. MBNL regulates alternative (orange boxes) splicing events, including cassette (e) exon, 5′ splice site, 3′ splice site, mutually exclusive exons, (i) intron retention [121,129] and alternative 3′ end formation by alternative cleavage and polyadenylation (pA) [122]. MBNL also regulates microRNA (miRNA) biogenesis [96], circular RNA (circRNA) formation [123], mRNA localization (horizontal arrow) [121] and increases mRNA stability (vertical arrow) [124]. All examples represent MBNL-mediated events and representative targeted RNAs are indicated.

In addition to CELF upregulation, current pathomechanistic models propose a major role for the MBNL family of RNA processing factors in DM1 and DM2 disease onset and progression. MBNL proteins are ~37-43 kDa *trans*-acting factors implicated in the alternative regulation of pre-mRNA splicing, pre-mRNA 3′-end cleavage/polyadenylation, mRNA localization, mRNA stability and microRNA biogenesis, as well as circular RNA generation during embryonic and postnatal development (Figure 4) [96,121,122,123,124,125,126,127]. Of the three mammalian *MBNL*/*Mbnl* paralogs, mouse *Mbnl1* and *Mbnl2* function primarily during the postnatal period to switch their RNA targets to adult expression patterns although they also play essential roles in utero since *Mbnl1*^−/−^; *Mbnl2*^−/−^ double knockout mice are embryonic lethal. On the other hand, *Mbnl3* is expressed primarily during embryonic development and during adult tissue regeneration [128].

**Figure 5:**
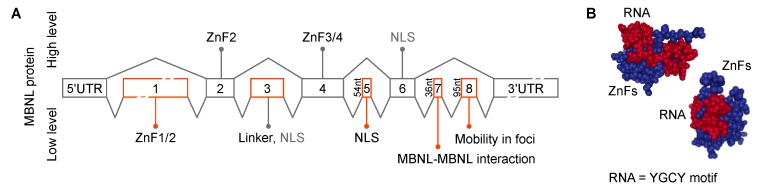
*MBNL* gene and ZnF structures. (**A**) Exonic structure and function of MBNL paralogs [135,148,149,150]. Exon enumeration is derived from previous studies [151]. ZnF1/2 and ZnF3/4–zinc finger domain pairs 1/2 and 3/4 respectively. NLS–nuclear localization signal. MBNL protein levels are shown as Low (bottom) and High (top) which trigger the indicated splicing events (black lines). (**B**) The structural model of MBNL1 zinc fingers (ZnF; blue) in complex with RNA (red) [152].

MBNL genes contain several alternatively spliced cassette exons important for RNA binding, splicing activity, nuclear localization and homotypic interactions (Figure 5A). MBNL proteins interact with their RNA targets via four zinc finger (ZnF) domains that bind GC steps mainly flanked by pyrimidines (Figure 5B) [130]. The consensus RNA binding site for MBNL proteins is YGCY, a repetitive motif in both CUG^exp^ and CCUG^exp^ RNAs [126,131,132,133]. The RNA processing activity of MBNL proteins is modulated by both the number and structural context of these binding motifs [132,134]. Expanded repeats in a single transcript may provide hundreds or even thousands, of high affinity MBNL binding sites (K_D_ = ~4–300 nM, depending on RNA structure/length and GC dinucleotide spacing) [135,136,137,138,139]. Expression of CUG^exp^ and CCUG^exp^ RNAs results in sequestration of the MBNL proteins in RNA foci (discussed in more detail below), depletion from the nucleoplasmic pool, the shift to more immature isoforms for MBNL targets and DM disease manifestations (Figure 3) [118]. In DM1, detection of >50 CTG repeats in blood is considered a molecular hallmark of the adult form of DM1 whereas >1000 CTGs greatly increases the risk of congenital DM1 (CDM) [140,141]. The possibility that CDM results from MBNL sequestration in utero has been recently tested using *Mbnl* conditional knockout mice. Interestingly, coordinate loss of *Mbnl1*, *Mbnl2* and *Mbnl3* expression in skeletal muscle is required to reproduce the congenital phenotype of respiratory muscle development [129]. MBNL loss-of-function has also been implicated in other CTG^exp^ diseases including Fuchs endothelial corneal dystrophy (FECD) [142,143] and spinocerebellar ataxia type 8 (SCA8) [144]. Finally, MBNL proteins may be indirectly sequestered by other RBPs or by other types of repeats, including CGG^exp^ associated with FXTAS and CAG^exp^ linked to polyglutamine diseases [145,146,147].

**Figure 6:**
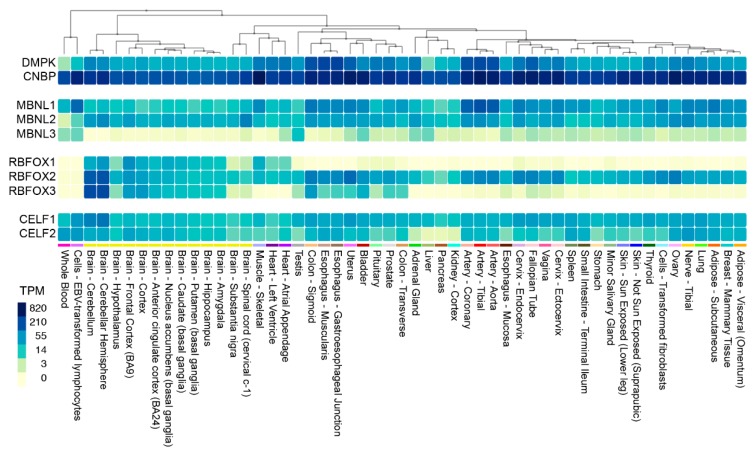
Expression of DM1 and DM2 relevant genes in human tissues. Heat map showing *DMPK*, *CNBP*, *MBNL1-3*, *RBFOX1-3* and *CELF1-2* gene expression in 53 unaffected tissues from >700 individuals. Data were obtained from the Genotype-Tissue Expression (GTEx) project website (gtexportal.org) and the heat map was generated using the Multi-Gene Query function (TPM, transcripts per million).

While DM1 and DM2 are classified as myotonic dystrophies characterized by myotonia, progressive myopathy and multiorgan involvement, they also have distinct clinical features [153]. DM2 is generally a later-onset disease with no congenital form and *CNBP* is expressed at much higher levels than *DMPK* (Figure 6). However, mis-splicing is usually less severe in DM2 compared to DM1 although the affinity of MBNL proteins for CCUG^exp^ is higher than CUG^exp^ [112,137,138]. What is the explanation for this discrepancy? Recently, RBFOX was shown to bind to CCUG^exp^ but not CUG^exp^, RNAs and accumulate in RNA foci suggesting that MBNL-RBFOX competition for RNA binding sites selectively reduces MBNL sequestration in DM2 to promote more adult-like splicing patterns [154]. Similar to CELF and MBNL, the RBFOX proteins are also expressed in multiple tissues (Figure 6). DM2 appears to have distinct cell-specific molecular signatures that differ from DM1, including no detectable MBNL1 sequestration in RNA foci and no mis-splicing of MBNL1 RNA targets in DM2 iPSC-derived cardiomyocytes [155]. Additionally, retention of CNBP intron 1 and nuclear export of the incompletely processed mRNA, occurs in multiple DM2 tissues suggesting that RAN translation of the intron 1 CCUG^exp^ results in enhanced expression of aberrant tetrapeptide repeats that are a major pathological factor in DM2 [58,156].

## 5. C(C)UG^exp^ RNA-MBNL Interactions in RNA Foci Formation

Evidence that RNA STR expansions form RNA foci was first shown just three years after the DM1 mutation discovery. Singer and colleagues reported unusual focal concentrations of expanded CUG repeats in DM1 nuclei visualized by RNA fluorescence in situ hybridization (RNA-FISH) using a fluorescent probe complementary to the expanded repeats [157]. Later, RNA foci were found in additional noncoding STR expansion diseases [158]. Since the discovery of RNA foci, our view of these structures has evolved from insoluble RNA aggregates into dynamic RNP complexes. RNA foci are non-membrane bound RNP complexes, a group that includes nucleoli, paraspeckles, nuclear speckles and Cajal bodies in the nucleus, as well as cytoplasmic P bodies, stress granules and neuronal and germ granules [159].

**Figure 7:**
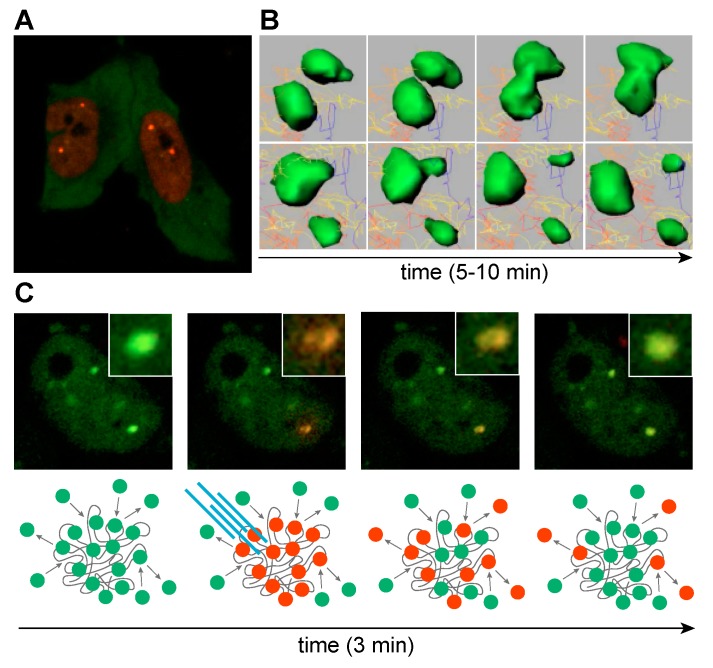
MBNL sequestration on expanded CUG^exp^ transcripts. (**A**) Life cell imaging of cells transfected with CUG^exp^, mCherry-MBNL1 and GFP-MBNL3. MBNL1 localizes primarily in nucleus, whereas MBNL3 is predominately cytoplasmic. (**B**) Time-lapse sequence images of CUG^exp^-GFP-MBNL1 complexes (green). Raw data were processed with Imaris software. (**C**) Time-lapse sequence images of a cell transiently transfected with CUG^exp^ and MBNL1 fused with Dendra2 fluorescence protein. MBNL1 accumulated in two distinct nuclear foci. Laser stimulated Dendra2-MBNL photoconversion from green (emission 507 nm) to red (emission 573 nm) in a single focus reveals dynamic exchange of MBNL between the focus and surrounding nucleoplasm in the course of minutes. Yellowish center of focus core might suggest that MBNL proteins localized in the focus core are less prone to exchange. Below each image, a scheme representing the experimental scheme. (**B**,**C**) Images adapted from Reference [135].

In DM1 and DM2, RNA foci are formed by co-transcriptional recruitment of MBNL proteins to C(C)UG^exp^ repeats (Figure 7A) [160]. The exact role of MBNL proteins in RNA foci assembly is not well understood but the size of RNA foci visualized by RNA-FISH increases at higher MBNL concentrations whereas protein depletion results in a decrease of foci size, suggesting a role for MBNL in promoting foci assembly and/or stability [135,160,161,162]. It is also possible that a single MBNL protein can interact with two RNA molecules because these proteins have two tandem ZnF pairs (Figure 5A) (ZnF 1/3 and ZnF 2/4 have similar motifs or CX_7_CX_6_CX_3_H and CX_7_CX_4_CX_3_H, X = amino acid, respectively) and a single ZnF pair is sufficient to bind to RNA (Figure 5B) [163]. RNA foci formation might also be enhanced by protein-protein interactions since homotypic interactions are mediated by the MBNL1 C-terminal region, which includes the alternatively spliced exon 7 [137,150,164]. Additional proteins have also been proposed to be sequestered within RNA foci (hnRNP H, H2, H3, F, A2/B1, K, L, DDX5, DDX17 and DHX9) but their role in foci formation is unclear [165]. Certainly, these other proteins could reside in foci, either through MBNL or C(C)UG^exp^ RNA interactions or by recruitment to existing MBNL-RNA^exp^ RBP complexes. These additional factors, including members of the DEAD-box family, could exacerbate or ameliorate the toxicity of these complexes by either promoting (e.g., DDX5) [166] or blocking (e.g., DDX6) [167] foci formation. Even though several other proteins have been detected within foci, including hnRNPs, their impact on the DM pathomechanism remains less clear [154,165]. It is also intriguing that RNP foci may be enriched in additional nuclear RNAs, which evokes the question of how the sequestration of non-C(C)UG^exp^ RNAs might contribute to disease.

The dynamic nature of RBP and expanded RNA interactions in foci has been reported for MBNL and RBFOX paralogs for various length STR RNAs [135,154,162,168]. RNP foci dynamics have been captured by live-cell imaging revealing that foci coalesce upon direct interaction or divide into smaller units (Figure 7B). Characteristics like foci number, shape and volume are prone to time-dependent and cell-state changes [135,162,169]. RNA foci may be stable for minutes or hours and while MBNL proteins are densely packed in foci, they dynamically translocate between RNA binding sites in foci and exchange with free MBNL proteins in the surrounding nucleoplasm (Figure 7C). Interestingly, a low level of expanded CUG RNA is readily saturated with MBNL proteins, which dynamically exchange with the unbound MBNL in the nucleoplasmic pool, while higher CUG loads severely deplete this pool [135]. Thus, RNA foci assembly in vivo is likely modulated not only by expansion repeat length but also by the spatiotemporal pattern of *DMPK* and *CNBP* expression. For instance, *DMPK* is expressed at a significantly higher level during fetal muscle development than in mature tissues and expression varies significantly between tissues [129].

## 6. Emerging Roles for RNA-RNA Multivalent Interactions in DM Disease

Early studies assessed the effects of RNA-MBNL interactions in the formation of RNA foci [170], however multivalent RNA-RNA interactions between expanded RNAs also impact foci formation. In transfected cells, overexpressed CUG, CAG and GGGGCC expanded RNAs form phase-separated gel-like droplets displaying ATP-dependent dynamics, implicating RNA-RNA interactions as major drivers of foci assembly [171]. However, it is not clear if CUG^exp^ and CCUG^exp^ expressed at endogenous levels promote RNA foci formation. For instance, *DMPK* transcripts are present at one to a few dozen molecules per cell in DM1 patient-derived myoblasts so RNA foci must be assembled from only a few transcripts [172,173].

Recently, a four-phase model was proposed whereby RNA-RNA, RNA-RBP and RBP-RBP interactions must reach an assembly threshold [159]. Based on this model, RNA foci assembly in DM1 and DM2 cells occurs when CUG^exp^ and CCUG^exp^ RNAs provide a scaffold for multivalent homotypic and heterotypic interactions that allow the formation of higher order assemblies. In CDM, with high mutant *DMPK* loads and relatively low MBNL expression, foci might be formed primarily through multivalent extra- and intramolecular interactions that are more prone to RNA gelation. In contrast, in DM1 mature skeletal muscles, CTG expansion size progressively increases while *DMPK* expression decreases and MBNL protein levels are relatively high.

## 7. Conclusions and Perspectives

Studies designed to elucidate the downstream pathways altered by the *DMPK* CTG^exp^ and *CNBP* CCTG^exp^ mutations in DM1 and DM2, respectively, have resulted in key insights into the developmental regulation of RNA alternative splicing and polyadenylation in the nucleus as well as RNA localization in the cytoplasm [118,119]. Early studies on DM1 also led to the discovery of nuclear RNA foci [157] while an investigation on SCA8 and DM1 revealed RAN translation [174] and both of these pathomechanisms have been subsequently described for other repeat expansion diseases [175,176]. Nevertheless, many outstanding questions remain including the possibility that a structure related to RNA foci may exist in unaffected cells and provide a critical additional step in nuclear RNA quality control.

## Abbreviations

3’UTR	3’ untranslated region
5’UTR	5’ untranslated region
ALS/FTD	Amyotrophic lateral sclerosis and frontotemporal dementia
C(C)UG^exp^	C(C)UG repeat expansion
CDM	Congenital myotonic dystrophy
DM1	Myotonic dystrophy type 1
DM2	Myotonic dystrophy type 2
FECD	Fuchs endothelial corneal dystrophy
FXTAS	Fragile X-associated tremor/ataxia syndrome
MBNL	Muscleblind-like
RAN	repeat-associated non-AUG
RBP	RNA-binding protein
RNP	Ribonucleoprotein
SCA	Spinocerebellar ataxia
STR	Short tandem repeat
ZnF	Zinc finger

## References

[1] Bourque G., Burns K.H., Gehring M., Gorbunova V., Seluanov A., Hammell M., Imbeault M., Izsvak Z., Levin H.L., Macfarlan T.S. (2018). Ten things you should know about transposable elements. Genome. Biol..

[2] Padeken J., Zeller P., Gasser S.M. (2015). Repeat DNA in genome organization and stability. Curr. Opin. Genet. Dev..

[3] McGinty R.J., Mirkin S.M. (2018). Cis- and trans-modifiers of repeat expansions: Blending model systems with human genetics. Trends Genet..

[4] López Castel A., Cleary J.D., Pearson C.E. (2010). Repeat instability as the basis for human diseases and as a potential target for therapy. Nat. Rev. Mol. Cell Biol.

[5] Iyer R.R., Pluciennik A., Napierala M., Wells R.D. (2015). DNA triplet repeat expansion and mismatch repair. Annu Rev. Biochem.

[6] LaCroix A.J., Stabley D., Sahraoui R., Adam M.P., Mehaffey M., Kernan K., Myers C.T., Fagerstrom C., Anadiotis G., Akkari Y.M. (2019). Ggc repeat expansion and exon 1 methylation of xylt1 is a common pathogenic variant in baratela-scott syndrome. Am. J. Hum. Genet..

[7] Lalioti M.D., Mirotsou M., Buresi C., Peitsch M.C., Rossier C., Ouazzani R., Baldy-Moulinier M., Bottani A., Malafosse A., Antonarakis S.E. (1997). Identification of mutations in cystatin b, the gene responsible for the unverricht-lundborg type of progressive myoclonus epilepsy (epm1). Am. J. Hum. Genet..

[8] van Kuilenburg A.B.P., Tarailo-Graovac M., Richmond P.A., Drögemöller B.I., Pouladi M.A., Leen R., Brand-Arzamendi K., Dobritzsch D., Dolzhenko E., Eberle M.A. (2019). Glutaminase deficiency caused by short tandem repeat expansion in. N Engl J. Med..

[9] Holmes S.E., O’Hearn E.E., McInnis M.G., Gorelick-Feldman D.A., Kleiderlein J.J., Callahan C., Kwak N.G., Ingersoll-Ashworth R.G., Sherr M., Sumner A.J. (1999). Expansion of a novel cag trinucleotide repeat in the 5’ region of ppp2r2b is associated with sca12. Nat. Genet..

[10] Gray S.J., Gerhardt J., Doerfler W., Small L.E., Fanning E. (2007). An origin of DNA replication in the promoter region of the human fragile x mental retardation (fmr1) gene. Mol. Cell Biol..

[11] Barasoain M., Barrenetxea G., Huerta I., Télez M., Criado B., Arrieta I. (2016). Study of the genetic etiology of primary ovarian insufficiency: Fmr1 gene. Genes (Basel).

[12] Knight S.J., Flannery A.V., Hirst M.C., Campbell L., Christodoulou Z., Phelps S.R., Pointon J., Middleton-Price H.R., Barnicoat A., Pembrey M.E. (1993). Trinucleotide repeat amplification and hypermethylation of a cpg island in fraxe mental retardation. Cell.

[13] Parrish J.E., Oostra B.A., Verkerk A.J., Richards C.S., Reynolds J., Spikes A.S., Shaffer L.G., Nelson D.L. (1994). Isolation of a gcc repeat showing expansion in fraxf, a fragile site distal to fraxa and fraxe. Nat. Genet..

[14] Metsu S., Rooms L., Rainger J., Taylor M.S., Bengani H., Wilson D.I., Chilamakuri C.S., Morrison H., Vandeweyer G., Reyniers E. (2014). Fra2a is a cgg repeat expansion associated with silencing of aff3. PLoS Genet..

[15] Metsu S., Rainger J.K., Debacker K., Bernhard B., Rooms L., Grafodatskaya D., Weksberg R., Fombonne E., Taylor M.S., Scherer S.W. (2014). A cgg-repeat expansion mutation in znf713 causes fra7a: Association with autistic spectrum disorder in two families. Hum. Mutat.

[16] Sarafidou T., Kahl C., Martinez-Garay I., Mangelsdorf M., Gesk S., Baker E., Kokkinaki M., Talley P., Maltby E.L., French L. (2004). Folate-sensitive fragile site fra10a is due to an expansion of a cgg repeat in a novel gene, fra10ac1, encoding a nuclear protein. Genomics.

[17] Debacker K., Winnepenninckx B., Longman C., Colgan J., Tolmie J., Murray R., van Luijk R., Scheers S., Fitzpatrick D., Kooy F. (2007). The molecular basis of the folate-sensitive fragile site fra11a at 11q13. Cytogenet Genome Res..

[18] Jones C., Penny L., Mattina T., Yu S., Baker E., Voullaire L., Langdon W.Y., Sutherland G.R., Richards R.I., Tunnacliffe A. (1995). Association of a chromosome deletion syndrome with a fragile site within the proto-oncogene cbl2. Nature.

[19] Winnepenninckx B., Debacker K., Ramsay J., Smeets D., Smits A., FitzPatrick D.R., Kooy R.F. (2007). Cgg-repeat expansion in the dip2b gene is associated with the fragile site fra12a on chromosome 12q13.1. Am. J. Hum. Genet..

[20] Nancarrow J.K., Kremer E., Holman K., Eyre H., Doggett N.A., Le Paslier D., Callen D.F., Sutherland G.R., Richards R.I. (1994). Implications of fra16a structure for the mechanism of chromosomal fragile site genesis. Science.

[21] Tian Y., Wang J.L., Huang W., Zeng S., Jiao B., Liu Z., Chen Z., Li Y., Wang Y., Min H.X. (2019). Expansion of human-specific ggc repeat in neuronal intranuclear inclusion disease-related disorders. Am. J. Hum. Genet..

[22] Renton A.E., Majounie E., Waite A., Simón-Sánchez J., Rollinson S., Gibbs J.R., Schymick J.C., Laaksovirta H., van Swieten J.C., Myllykangas L. (2011). A hexanucleotide repeat expansion in c9orf72 is the cause of chromosome 9p21-linked als-ftd. Neuron.

[23] DeJesus-Hernandez M., Mackenzie I.R., Boeve B.F., Boxer A.L., Baker M., Rutherford N.J., Nicholson A.M., Finch N.A., Flynn H., Adamson J. (2011). Expanded ggggcc hexanucleotide repeat in noncoding region of c9orf72 causes chromosome 9p-linked ftd and als. Neuron.

[24] Ishiura H., Doi K., Mitsui J., Yoshimura J., Matsukawa M.K., Fujiyama A., Toyoshima Y., Kakita A., Takahashi H., Suzuki Y. (2018). Expansions of intronic tttca and tttta repeats in benign adult familial myoclonic epilepsy. Nat. Genet..

[25] Cortese A., Simone R., Sullivan R., Vandrovcova J., Tariq H., Yau W.Y., Humphrey J., Jaunmuktane Z., Sivakumar P., Polke J. (2019). Biallelic expansion of an intronic repeat in rfc1 is a common cause of late-onset ataxia. Nat. Genet..

[26] Liquori C.L., Ricker K., Moseley M.L., Jacobsen J.F., Kress W., Naylor S.L., Day J.W., Ranum L.P. (2001). Myotonic dystrophy type 2 caused by a cctg expansion in intron 1 of znf9. Science.

[27] Wieben E.D., Aleff R.A., Tosakulwong N., Butz M.L., Highsmith W.E., Edwards A.O., Baratz K.H. (2012). A common trinucleotide repeat expansion within the transcription factor 4 (tcf4, e2-2) gene predicts fuchs corneal dystrophy. PLoS ONE.

[28] Campuzano V., Montermini L., Moltò M.D., Pianese L., Cossée M., Cavalcanti F., Monros E., Rodius F., Duclos F., Monticelli A. (1996). Friedreich’s ataxia: Autosomal recessive disease caused by an intronic gaa triplet repeat expansion. Science.

[29] Matsuura T., Yamagata T., Burgess D.L., Rasmussen A., Grewal R.P., Watase K., Khajavi M., McCall A.E., Davis C.F., Zu L. (2000). Large expansion of the attct pentanucleotide repeat in spinocerebellar ataxia type 10. Nat. Genet..

[30] Sato N., Amino T., Kobayashi K., Asakawa S., Ishiguro T., Tsunemi T., Takahashi M., Matsuura T., Flanigan K.M., Iwasaki S. (2009). Spinocerebellar ataxia type 31 is associated with "inserted" penta-nucleotide repeats containing (tggaa)n. Am. J. Hum. Genet..

[31] Kobayashi H., Abe K., Matsuura T., Ikeda Y., Hitomi T., Akechi Y., Habu T., Liu W., Okuda H., Koizumi A. (2011). Expansion of intronic ggcctg hexanucleotide repeat in nop56 causes sca36, a type of spinocerebellar ataxia accompanied by motor neuron involvement. Am. J. Hum. Genet..

[32] Seixas A.I., Loureiro J.R., Costa C., Ordóñez-Ugalde A., Marcelino H., Oliveira C.L., Loureiro J.L., Dhingra A., Brandão E., Cruz V.T. (2017). A pentanucleotide atttc repeat insertion in the non-coding region of dab1, mapping to sca37, causes spinocerebellar ataxia. Am. J. Hum. Genet..

[33] Bragg D.C., Mangkalaphiban K., Vaine C.A., Kulkarni N.J., Shin D., Yadav R., Dhakal J., Ton M.L., Cheng A., Russo C.T. (2017). Disease onset in x-linked dystonia-parkinsonism correlates with expansion of a hexameric repeat within an sva retrotransposon in. Proc. Natl. Acad. Sci. USA.

[34] Koide R., Ikeuchi T., Onodera O., Tanaka H., Igarashi S., Endo K., Takahashi H., Kondo R., Ishikawa A., Hayashi T. (1994). Unstable expansion of cag repeat in hereditary dentatorubral-pallidoluysian atrophy (drpla). Nat. Genet..

[35] MacDonald M.E., Ambrose C.M., Duyao M.P., Myers R.H., Lin C., Srinidhi L., Barnes G., Taylor S.A., James M., Groot N. (1993). A novel gene containing a trinucleotide repeat that is expanded and unstable on huntington’s disease chromosomes. The huntington’s disease collaborative research group. Cell.

[36] La Spada A.R., Wilson E.M., Lubahn D.B., Harding A.E., Fischbeck K.H. (1991). Androgen receptor gene mutations in x-linked spinal and bulbar muscular atrophy. Nature.

[37] Orr H.T., Chung M.Y., Banfi S., Kwiatkowski T.J., Servadio A., Beaudet A.L., McCall A.E., Duvick L.A., Ranum L.P., Zoghbi H.Y. (1993). Expansion of an unstable trinucleotide cag repeat in spinocerebellar ataxia type 1. Nat. Genet..

[38] Sanpei K., Takano H., Igarashi S., Sato T., Oyake M., Sasaki H., Wakisaka A., Tashiro K., Ishida Y., Ikeuchi T. (1996). Identification of the spinocerebellar ataxia type 2 gene using a direct identification of repeat expansion and cloning technique, direct. Nat. Genet..

[39] Stevanin G., Le Guern E., Ravisé N., Chneiweiss H., Dürr A., Cancel G., Vignal A., Boch A.L., Ruberg M., Penet C. (1994). A third locus for autosomal dominant cerebellar ataxia type i maps to chromosome 14q24.3-qter: Evidence for the existence of a fourth locus. Am. J. Hum. Genet..

[40] Jodice C., Mantuano E., Veneziano L., Trettel F., Sabbadini G., Calandriello L., Francia A., Spadaro M., Pierelli F., Salvi F. (1997). Episodic ataxia type 2 (ea2) and spinocerebellar ataxia type 6 (sca6) due to cag repeat expansion in the cacna1a gene on chromosome 19p. Hum. Mol. Genet..

[41] David G., Dürr A., Stevanin G., Cancel G., Abbas N., Benomar A., Belal S., Lebre A.S., Abada-Bendib M., Grid D. (1998). Molecular and clinical correlations in autosomal dominant cerebellar ataxia with progressive macular dystrophy (sca7). Hum. Mol. Genet..

[42] Koob M.D., Moseley M.L., Schut L.J., Benzow K.A., Bird T.D., Day J.W., Ranum L.P. (1999). An untranslated ctg expansion causes a novel form of spinocerebellar ataxia (sca8). Nat. Genet..

[43] Nakamura K., Jeong S.Y., Uchihara T., Anno M., Nagashima K., Nagashima T., Ikeda S., Tsuji S., Kanazawa I. (2001). Sca17, a novel autosomal dominant cerebellar ataxia caused by an expanded polyglutamine in tata-binding protein. Hum. Mol. Genet..

[44] De Baere E., Beysen D., Oley C., Lorenz B., Cocquet J., De Sutter P., Devriendt K., Dixon M., Fellous M., Fryns J.P. (2003). Foxl2 and bpes: Mutational hotspots, phenotypic variability and revision of the genotype-phenotype correlation. Am. J. Hum. Genet..

[45] Mundlos S., Otto F., Mundlos C., Mulliken J.B., Aylsworth A.S., Albright S., Lindhout D., Cole W.G., Henn W., Knoll J.H. (1997). Mutations involving the transcription factor cbfa1 cause cleidocranial dysplasia. Cell.

[46] Amiel J., Laudier B., Attié-Bitach T., Trang H., de Pontual L., Gener B., Trochet D., Etchevers H., Ray P., Simonneau M. (2003). Polyalanine expansion and frameshift mutations of the paired-like homeobox gene phox2b in congenital central hypoventilation syndrome. Nat. Genet..

[47] Goodman F.R., Bacchelli C., Brady A.F., Brueton L.A., Fryns J.P., Mortlock D.P., Innis J.W., Holmes L.B., Donnenfeld A.E., Feingold M. (2000). Novel hoxa13 mutations and the phenotypic spectrum of hand-foot-genital syndrome. Am. J. Hum. Genet..

[48] Brown S.A., Warburton D., Brown L.Y., Yu C.Y., Roeder E.R., Stengel-Rutkowski S., Hennekam R.C., Muenke M. (1998). Holoprosencephaly due to mutations in zic2, a homologue of drosophila odd-paired. Nat. Genet..

[49] Brais B., Bouchard J.P., Xie Y.G., Rochefort D.L., Chrétien N., Tomé F.M., Lafrenière R.G., Rommens J.M., Uyama E., Nohira O. (1998). Short gcg expansions in the pabp2 gene cause oculopharyngeal muscular dystrophy. Nat. Genet..

[50] Muragaki Y., Mundlos S., Upton J., Olsen B.R. (1996). Altered growth and branching patterns in synpolydactyly caused by mutations in hoxd13. Science.

[51] Kato M., Das S., Petras K., Kitamura K., Morohashi K., Abuelo D.N., Barr M., Bonneau D., Brady A.F., Carpenter N.J. (2004). Mutations of arx are associated with striking pleiotropy and consistent genotype-phenotype correlation. Hum. Mutat.

[52] Strømme P., Mangelsdorf M.E., Shaw M.A., Lower K.M., Lewis S.M., Bruyere H., Lütcherath V., Gedeon A.K., Wallace R.H., Scheffer I.E. (2002). Mutations in the human ortholog of aristaless cause x-linked mental retardation and epilepsy. Nat. Genet..

[53] Laumonnier F., Ronce N., Hamel B.C., Thomas P., Lespinasse J., Raynaud M., Paringaux C., Van Bokhoven H., Kalscheuer V., Fryns J.P. (2002). Transcription factor sox3 is involved in x-linked mental retardation with growth hormone deficiency. Am. J. Hum. Genet..

[54] Délot E., King L.M., Briggs M.D., Wilcox W.R., Cohn D.H. (1999). Trinucleotide expansion mutations in the cartilage oligomeric matrix protein (comp) gene. Hum. Mol. Genet..

[55] Mahadevan M., Tsilfidis C., Sabourin L., Shutler G., Amemiya C., Jansen G., Neville C., Narang M., Barceló J., O’Hoy K. (1992). Myotonic dystrophy mutation: An unstable ctg repeat in the 3’ untranslated region of the gene. Science.

[56] Holmes S.E., O’Hearn E., Rosenblatt A., Callahan C., Hwang H.S., Ingersoll-Ashworth R.G., Fleisher A., Stevanin G., Brice A., Potter N.T. (2001). A repeat expansion in the gene encoding junctophilin-3 is associated with huntington disease-like 2. Nat. Genet..

[57] Neueder A., Landles C., Ghosh R., Howland D., Myers R.H., Faull R.L.M., Tabrizi S.J., Bates G.P. (2017). The pathogenic exon 1 htt protein is produced by incomplete splicing in huntington’s disease patients. Sci. Rep..

[58] Sznajder Ł.J., Thomas J.D., Carrell E.M., Reid T., McFarland K.N., Cleary J.D., Oliveira R., Nutter C.A., Bhatt K., Sobczak K. (2018). Intron retention induced by microsatellite expansions as a disease biomarker. Proc. Natl. Acad. Sci. USA.

[59] Batra R., Charizanis K., Swanson M.S. (2010). Partners in crime: Bidirectional transcription in unstable microsatellite disease. Hum. Mol. Genet..

[60] Mohan A., Goodwin M., Swanson M.S. (2014). Rna-protein interactions in unstable microsatellite diseases. Brain Res..

[61] Szlachcic W.J., Switonski P.M., Kurkowiak M., Wiatr K., Figiel M. (2015). Mouse polyq database: A new online resource for research using mouse models of neurodegenerative diseases. Mol. Brain.

[62] Amiel J., Trochet D., Clément-Ziza M., Munnich A., Lyonnet S. (2004). Polyalanine expansions in human. Hum. Mol. Genet..

[63] Cleary J.D., Pattamatta A., Ranum L.P.W. (2018). Repeat-associated non-atg (ran) translation. J. Biol Chem.

[64] Taylor J.P., Brown R.H., Cleveland D.W. (2016). Decoding als: From genes to mechanism. Nature.

[65] Polak U., Li Y., Butler J.S., Napierala M. (2016). Alleviating gaa repeat induced transcriptional silencing of the friedreich’s ataxia gene during somatic cell reprogramming. Stem Cells Dev..

[66] Liu X.S., Wu H., Krzisch M., Wu X., Graef J., Muffat J., Hnisz D., Li C.H., Yuan B., Xu C. (2018). Rescue of fragile x syndrome neurons by DNA methylation editing of the fmr1 gene. Cell.

[67] Leroux A.E., Schulze J.O., Biondi R.M. (2018). Agc kinases, mechanisms of regulation and innovative drug development. Semin Cancer Biol..

[68] Oude Ophuis R.J., Mulders S.A., van Herpen R.E., van de Vorstenbosch R., Wieringa B., Wansink D.G. (2009). Dmpk protein isoforms are differentially expressed in myogenic and neural cell lineages. Muscle Nerve.

[69] Benhalevy D., Gupta S.K., Danan C.H., Ghosal S., Sun H.W., Kazemier H.G., Paeschke K., Hafner M., Juranek S.A. (2017). The human cchc-type zinc finger nucleic acid-binding protein binds g-rich elements in target mrna coding sequences and promotes translation. Cell Rep..

[70] Reddy S., Smith D.B., Rich M.M., Leferovich J.M., Reilly P., Davis B.M., Tran K., Rayburn H., Bronson R., Cros D. (1996). Mice lacking the myotonic dystrophy protein kinase develop a late onset progressive myopathy. Nat. Genet..

[71] Jansen G., Groenen P.J., Bachner D., Jap P.H., Coerwinkel M., Oerlemans F., van den Broek W., Gohlsch B., Pette D., Plomp J.J. (1996). Abnormal myotonic dystrophy protein kinase levels produce only mild myopathy in mice. Nat. Genet..

[72] Carrell S.T., Carrell E.M., Auerbach D., Pandey S.K., Bennett C.F., Dirksen R.T., Thornton C.A. (2016). Dmpk gene deletion or antisense knockdown does not compromise cardiac or skeletal muscle function in mice. Hum. Mol. Genet..

[73] Santoro M., Fontana L., Maiorca F., Centofanti F., Massa R., Silvestri G., Novelli G., Botta A. (2018). Expanded [cctg]n repetitions are not associated with abnormal methylation at the cnbp locus in myotonic dystrophy type 2 (dm2) patients. Biochim. Biophys. Acta Mol. Basis Dis..

[74] Margolis J.M., Schoser B.G., Moseley M.L., Day J.W., Ranum L.P. (2006). Dm2 intronic expansions: Evidence for ccug accumulation without flanking sequence or effects on znf9 mrna processing or protein expression. Hum. Mol. Genet..

[75] Botta A., Caldarola S., Vallo L., Bonifazi E., Fruci D., Gullotta F., Massa R., Novelli G., Loreni F. (2006). Effect of the [cctg]n repeat expansion on znf9 expression in myotonic dystrophy type ii (dm2). Biochim. Biophys. Acta.

[76] Massa R., Panico M.B., Caldarola S., Fusco F.R., Sabatelli P., Terracciano C., Botta A., Novelli G., Bernardi G., Loreni F. (2010). The myotonic dystrophy type 2 (dm2) gene product zinc finger protein 9 (znf9) is associated with sarcomeres and normally localized in dm2 patients’ muscles. Neuropathol Appl. Neurobiol..

[77] Raheem O., Olufemi S.E., Bachinski L.L., Vihola A., Sirito M., Holmlund-Hampf J., Haapasalo H., Li Y.P., Udd B., Krahe R. (2010). Mutant (cctg)n expansion causes abnormal expression of zinc finger protein 9 (znf9) in myotonic dystrophy type 2. Am. J. Pathol..

[78] Huichalaf C., Schoser B., Schneider-Gold C., Jin B., Sarkar P., Timchenko L. (2009). Reduction of the rate of protein translation in patients with myotonic dystrophy 2. J. Neurosci.

[79] Pelletier R., Hamel F., Beaulieu D., Patry L., Haineault C., Tarnopolsky M., Schoser B., Puymirat J. (2009). Absence of a differentiation defect in muscle satellite cells from dm2 patients. Neurobiol. Dis..

[80] Wei C., Stock L., Schneider-Gold C., Sommer C., Timchenko N.A., Timchenko L. (2018). Reduction of cellular nucleic acid binding protein encoded by a myotonic dystrophy type 2 gene causes muscle atrophy. Mol. Cell Biol..

[81] Mankodi A., Takahashi M.P., Jiang H., Beck C.L., Bowers W.J., Moxley R.T., Cannon S.C., Thornton C.A. (2002). Expanded cug repeats trigger aberrant splicing of clc-1 chloride channel pre-mrna and hyperexcitability of skeletal muscle in myotonic dystrophy. Mol. Cell.

[82] Charlet-B N., Savkur R.S., Singh G., Philips A.V., Grice E.A., Cooper T.A. (2002). Loss of the muscle-specific chloride channel in type 1 myotonic dystrophy due to misregulated alternative splicing. Mol. Cell.

[83] Wheeler T.M., Sobczak K., Lueck J.D., Osborne R.J., Lin X., Dirksen R.T., Thornton C.A. (2009). Reversal of rna dominance by displacement of protein sequestered on triplet repeat rna. Science.

[84] Tang Z.Z., Yarotskyy V., Wei L., Sobczak K., Nakamori M., Eichinger K., Moxley R.T., Dirksen R.T., Thornton C.A. (2012). Muscle weakness in myotonic dystrophy associated with misregulated splicing and altered gating of ca(v)1.1 calcium channel. Hum. Mol. Genet..

[85] Fugier C., Klein A.F., Hammer C., Vassilopoulos S., Ivarsson Y., Toussaint A., Tosch V., Vignaud A., Ferry A., Messaddeq N. (2011). Misregulated alternative splicing of bin1 is associated with t tubule alterations and muscle weakness in myotonic dystrophy. Nat. Med..

[86] Rau F., Lainé J., Ramanoudjame L., Ferry A., Arandel L., Delalande O., Jollet A., Dingli F., Lee K.Y., Peccate C. (2015). Abnormal splicing switch of dmd’s penultimate exon compromises muscle fibre maintenance in myotonic dystrophy. Nat. Commun.

[87] Tang Y., Wang H., Wei B., Guo Y., Gu L., Yang Z., Zhang Q., Wu Y., Yuan Q., Zhao G. (2015). Cug-bp1 regulates ryr1 asi alternative splicing in skeletal muscle atrophy. Sci. Rep..

[88] Gao Z., Cooper T.A. (2013). Reexpression of pyruvate kinase m2 in type 1 myofibers correlates with altered glucose metabolism in myotonic dystrophy. Proc. Natl. Acad. Sci. USA.

[89] Freyermuth F., Rau F., Kokunai Y., Linke T., Sellier C., Nakamori M., Kino Y., Arandel L., Jollet A., Thibault C. (2016). Splicing misregulation of scn5a contributes to cardiac-conduction delay and heart arrhythmia in myotonic dystrophy. Nat. Commun..

[90] Pang P.D., Alsina K.M., Cao S., Koushik A.B., Wehrens X.H.T., Cooper T.A. (2018). Crispr -mediated expression of the fetal scn5a isoform in adult mice causes conduction defects and arrhythmias. J. Am. Heart Assoc..

[91] Philips A.V., Timchenko L.T., Cooper T.A. (1998). Disruption of splicing regulated by a cug-binding protein in myotonic dystrophy. Science.

[92] Sergeant N., Sablonnière B., Schraen-Maschke S., Ghestem A., Maurage C.A., Wattez A., Vermersch P., Delacourte A. (2001). Dysregulation of human brain microtubule-associated tau mrna maturation in myotonic dystrophy type 1. Hum. Mol. Genet..

[93] Jiang H., Mankodi A., Swanson M.S., Moxley R.T., Thornton C.A. (2004). Myotonic dystrophy type 1 is associated with nuclear foci of mutant rna, sequestration of muscleblind proteins and deregulated alternative splicing in neurons. Hum. Mol. Genet..

[94] Savkur R.S., Philips A.V., Cooper T.A. (2001). Aberrant regulation of insulin receptor alternative splicing is associated with insulin resistance in myotonic dystrophy. Nat. Genet..

[95] Renna L.V., Bosè F., Iachettini S., Fossati B., Saraceno L., Milani V., Colombo R., Meola G., Cardani R. (2017). Receptor and post-receptor abnormalities contribute to insulin resistance in myotonic dystrophy type 1 and type 2 skeletal muscle. PLoS ONE.

[96] Rau F., Freyermuth F., Fugier C., Villemin J.P., Fischer M.C., Jost B., Dembele D., Gourdon G., Nicole A., Duboc D. (2011). Misregulation of mir-1 processing is associated with heart defects in myotonic dystrophy. Nat. Struct Mol. Biol..

[97] Chen W., Liang Y., Deng W., Shimizu K., Ashique A.M., Li E., Li Y.P. (2003). The zinc-finger protein cnbp is required for forebrain formation in the mouse. Development.

[98] Chen W., Wang Y., Abe Y., Cheney L., Udd B., Li Y.P. (2007). Haploinsuffciency for znf9 in znf9+/- mice is associated with multiorgan abnormalities resembling myotonic dystrophy. J. Mol. Biol.

[99] de Peralta M.S., Mouguelar V.S., Sdrigotti M.A., Ishiy F.A., Fanganiello R.D., Passos-Bueno M.R., Coux G., Calcaterra N.B. (2016). Cnbp ameliorates treacher collins syndrome craniofacial anomalies through a pathway that involves redox-responsive genes. Cell Death Dis..

[100] Mankodi A., Logigian E., Callahan L., McClain C., White R., Henderson D., Krym M., Thornton C.A. (2000). Myotonic dystrophy in transgenic mice expressing an expanded cug repeat. Science.

[101] Handa V., Yeh H.J., McPhie P., Usdin K. (2005). The auucu repeats responsible for spinocerebellar ataxia type 10 form unusual rna hairpins. J. Biol. Chem..

[102] Park H., González À., Yildirim I., Tran T., Lohman J.R., Fang P., Guo M., Disney M.D. (2015). Crystallographic and computational analyses of auucu repeating rna that causes spinocerebellar ataxia type 10 (sca10). Biochemistry.

[103] Napierała M., Krzyzosiak W.J. (1997). Cug repeats present in myotonin kinase rna form metastable “slippery” hairpins. J. Biol. Chem..

[104] Childs-Disney J.L., Yildirim I., Park H., Lohman J.R., Guan L., Tran T., Sarkar P., Schatz G.C., Disney M.D. (2014). Structure of the myotonic dystrophy type 2 rna and designed small molecules that reduce toxicity. ACS Chem. Biol..

[105] Zhang Y., Roland C., Sagui C. (2018). Structural and dynamical characterization of DNA and rna quadruplexes obtained from the ggggcc and gggcct hexanucleotide repeats associated with c9ftd/als and sca36 diseases. ACS Chem. Neurosci..

[106] Jansen G., Willems P., Coerwinkel M., Nillesen W., Smeets H., Vits L., Höweler C., Brunner H., Wieringa B. (1994). Gonosomal mosaicism in myotonic dystrophy patients: Involvement of mitotic events in (ctg)n repeat variation and selection against extreme expansion in sperm. Am. J. Hum. Genet..

[107] Wong L.J., Ashizawa T., Monckton D.G., Caskey C.T., Richards C.S. (1995). Somatic heterogeneity of the ctg repeat in myotonic dystrophy is age and size dependent. Am. J. Hum. Genet..

[108] Lia A.S., Seznec H., Hofmann-Radvanyi H., Radvanyi F., Duros C., Saquet C., Blanche M., Junien C., Gourdon G. (1998). Somatic instability of the ctg repeat in mice transgenic for the myotonic dystrophy region is age dependent but not correlated to the relative intertissue transcription levels and proliferative capacities. Hum. Mol. Genet..

[109] Monckton D.G., Coolbaugh M.I., Ashizawa K.T., Siciliano M.J., Caskey C.T. (1997). Hypermutable myotonic dystrophy ctg repeats in transgenic mice. Nat. Genet..

[110] Thornton C.A., Johnson K., Moxley R.T. (1994). Myotonic dystrophy patients have larger ctg expansions in skeletal muscle than in leukocytes. Ann. Neurol.

[111] Zatz M., Passos-Bueno M.R., Cerqueira A., Marie S.K., Vainzof M., Pavanello R.C. (1995). Analysis of the ctg repeat in skeletal muscle of young and adult myotonic dystrophy patients: When does the expansion occur?. Hum. Mol. Genet..

[112] Nakamori M., Sobczak K., Puwanant A., Welle S., Eichinger K., Pandya S., Dekdebrun J., Heatwole C.R., McDermott M.P., Chen T. (2013). Splicing biomarkers of disease severity in myotonic dystrophy. Ann. Neurol..

[113] Meola G., Cardani R. (2017). Myotonic dystrophy type 2 and modifier genes: An update on clinical and pathomolecular aspects. Neurol. Sci..

[114] Braz S.O., Acquaire J., Gourdon G., Gomes-Pereira M. (2018). Of mice and men: Advances in the understanding of neuromuscular aspects of myotonic dystrophy. Front. Neurol..

[115] Matloka M., Klein A.F., Rau F., Furling D. (2018). Cells of matter-in vitro models for myotonic dystrophy. Front. Neurol..

[116] Kuyumcu-Martinez N.M., Wang G.S., Cooper T.A. (2007). Increased steady-state levels of cugbp1 in myotonic dystrophy 1 are due to pkc-mediated hyperphosphorylation. Mol. Cell.

[117] Salisbury E., Schoser B., Schneider-Gold C., Wang G.L., Huichalaf C., Jin B., Sirito M., Sarkar P., Krahe R., Timchenko N.A. (2009). Expression of rna ccug repeats dysregulates translation and degradation of proteins in myotonic dystrophy 2 patients. Am. J. Pathol.

[118] Goodwin M., Swanson M.S. (2014). Rna-binding protein misregulation in microsatellite expansion disorders. Adv. Exp. Med. Biol..

[119] Brinegar A.E., Cooper T.A. (2016). Roles for rna-binding proteins in development and disease. Brain Res..

[120] Lin X., Miller J.W., Mankodi A., Kanadia R.N., Yuan Y., Moxley R.T., Swanson M.S., Thornton C.A. (2006). Failure of mbnl1-dependent post-natal splicing transitions in myotonic dystrophy. Hum. Mol. Genet..

[121] Wang E.T., Cody N.A., Jog S., Biancolella M., Wang T.T., Treacy D.J., Luo S., Schroth G.P., Housman D.E., Reddy S. (2012). Transcriptome-wide regulation of pre-mrna splicing and mrna localization by muscleblind proteins. Cell.

[122] Batra R., Charizanis K., Manchanda M., Mohan A., Li M., Finn D.J., Goodwin M., Zhang C., Sobczak K., Thornton C.A. (2014). Loss of mbnl leads to disruption of developmentally regulated alternative polyadenylation in rna-mediated disease. Mol. Cell.

[123] Ashwal-Fluss R., Meyer M., Pamudurti N.R., Ivanov A., Bartok O., Hanan M., Evantal N., Memczak S., Rajewsky N., Kadener S. (2014). Circrna biogenesis competes with pre-mrna splicing. Mol. Cell.

[124] Masuda A., Andersen H.S., Doktor T.K., Okamoto T., Ito M., Andresen B.S., Ohno K. (2012). Cugbp1 and mbnl1 preferentially bind to 3’ utrs and facilitate mrna decay. Sci. Rep..

[125] Goodwin M., Mohan A., Batra R., Lee K.Y., Charizanis K., Fernandez Gomez F.J., Eddarkaoui S., Sergeant N., Buee L., Kimura T. (2015). Mbnl sequestration by toxic rnas and rna misprocessing in the myotonic dystrophy brain. Cell Rep..

[126] Du H., Cline M.S., Osborne R.J., Tuttle D.L., Clark T.A., Donohue J.P., Hall M.P., Shiue L., Swanson M.S., Thornton C.A. (2010). Aberrant alternative splicing and extracellular matrix gene expression in mouse models of myotonic dystrophy. Nat. Struct Mol. Biol.

[127] Perbellini R., Greco S., Sarra-Ferraris G., Cardani R., Capogrossi M.C., Meola G., Martelli F. (2011). Dysregulation and cellular mislocalization of specific mirnas in myotonic dystrophy type 1. Neuromuscul Disord.

[128] Kanadia R.N., Urbinati C.R., Crusselle V.J., Luo D., Lee Y.J., Harrison J.K., Oh S.P., Swanson M.S. (2003). Developmental expression of mouse muscleblind genes mbnl1, mbnl2 and mbnl3. Gene Expr. Patterns.

[129] Thomas J.D., Sznajder Ł.J., Bardhi O., Aslam F.N., Anastasiadis Z.P., Scotti M.M., Nishino I., Nakamori M., Wang E.T., Swanson M.S. (2017). Disrupted prenatal rna processing and myogenesis in congenital myotonic dystrophy. Genes Dev..

[130] Teplova M., Patel D.J. (2008). Structural insights into rna recognition by the alternative-splicing regulator muscleblind-like mbnl1. Nat. Struct Mol. Biol..

[131] Zhang C., Lee K.Y., Swanson M.S., Darnell R.B. (2013). Prediction of clustered rna-binding protein motif sites in the mammalian genome. Nucleic Acids Res..

[132] Goers E.S., Purcell J., Voelker R.B., Gates D.P., Berglund J.A. (2010). Mbnl1 binds gc motifs embedded in pyrimidines to regulate alternative splicing. Nucleic Acids Res..

[133] Charizanis K., Lee K.Y., Batra R., Goodwin M., Zhang C., Yuan Y., Shiue L., Cline M., Scotti M.M., Xia G. (2012). Muscleblind-like 2-mediated alternative splicing in the developing brain and dysregulation in myotonic dystrophy. Neuron.

[134] Taylor K., Sznajder L.J., Cywoniuk P., Thomas J.D., Swanson M.S., Sobczak K. (2018). Mbnl splicing activity depends on rna binding site structural context. Nucleic Acids Res..

[135] Sznajder Ł.J., Michalak M., Taylor K., Cywoniuk P., Kabza M., Wojtkowiak-Szlachcic A., Matloka M., Konieczny P., Sobczak K. (2016). Mechanistic determinants of mbnl activity. Nucleic Acids Res..

[136] deLorimier E., Coonrod L.A., Copperman J., Taber A., Reister E.E., Sharma K., Todd P.K., Guenza M.G., Berglund J.A. (2014). Modifications to toxic cug rnas induce structural stability, rescue mis-splicing in a myotonic dystrophy cell model and reduce toxicity in a myotonic dystrophy zebrafish model. Nucleic Acids Res..

[137] Yuan Y., Compton S.A., Sobczak K., Stenberg M.G., Thornton C.A., Griffith J.D., Swanson M.S. (2007). Muscleblind-like 1 interacts with rna hairpins in splicing target and pathogenic rnas. Nucleic Acids Res..

[138] Warf M.B., Berglund J.A. (2007). Mbnl binds similar rna structures in the cug repeats of myotonic dystrophy and its pre-mrna substrate cardiac troponin t. RNA.

[139] Cass D., Hotchko R., Barber P., Jones K., Gates D.P., Berglund J.A. (2011). The four zn fingers of mbnl1 provide a flexible platform for recognition of its rna binding elements. BMC Mol. Biol.

[140] Joseph J.T., Richards C.S., Anthony D.C., Upton M., Perez-Atayde A.R., Greenstein P. (1997). Congenital myotonic dystrophy pathology and somatic mosaicism. Neurology.

[141] Tsilfidis C., MacKenzie A.E., Mettler G., Barceló J., Korneluk R.G. (1992). Correlation between ctg trinucleotide repeat length and frequency of severe congenital myotonic dystrophy. Nat. Genet..

[142] Du J., Aleff R.A., Soragni E., Kalari K., Nie J., Tang X., Davila J., Kocher J.P., Patel S.V., Gottesfeld J.M. (2015). Rna toxicity and missplicing in the common eye disease fuchs endothelial corneal dystrophy. J. Biol. Chem..

[143] Wieben E.D., Aleff R.A., Tang X., Butz M.L., Kalari K.R., Highsmith E.W., Jen J., Vasmatzis G., Patel S.V., Maguire L.J. (2017). Trinucleotide repeat expansion in the transcription factor 4 (tcf4) gene leads to widespread mrna splicing changes in fuchs’ endothelial corneal dystrophy. Invest. Ophthalmol Vis. Sci.

[144] Daughters R.S., Tuttle D.L., Gao W., Ikeda Y., Moseley M.L., Ebner T.J., Swanson M.S., Ranum L.P. (2009). Rna gain-of-function in spinocerebellar ataxia type 8. PLoS Genet..

[145] Mykowska A., Sobczak K., Wojciechowska M., Kozlowski P., Krzyzosiak W.J. (2011). Cag repeats mimic cug repeats in the misregulation of alternative splicing. Nucleic Acids Res..

[146] Kumar A., Parkesh R., Sznajder L.J., Childs-Disney J.L., Sobczak K., Disney M.D. (2012). Chemical correction of pre-mrna splicing defects associated with sequestration of muscleblind-like 1 protein by expanded r(cag)-containing transcripts. ACS Chem. Biol..

[147] Sellier C., Rau F., Liu Y., Tassone F., Hukema R.K., Gattoni R., Schneider A., Richard S., Willemsen R., Elliott D.J. (2010). Sam68 sequestration and partial loss of function are associated with splicing alterations in fxtas patients. Embo J..

[148] Konieczny P., Stepniak-Konieczna E., Sobczak K. (2018). Mbnl expression in autoregulatory feedback loops. RNA Biol..

[149] Kino Y., Washizu C., Kurosawa M., Oma Y., Hattori N., Ishiura S., Nukina N. (2015). Nuclear localization of mbnl1: Splicing-mediated autoregulation and repression of repeat-derived aberrant proteins. Hum. Mol. Genet..

[150] Tabaglio T., Low D.H., Teo W.K.L., Goy P.A., Cywoniuk P., Wollmann H., Ho J., Tan D., Aw J., Pavesi A. (2018). Mbnl1 alternative splicing isoforms play opposing roles in cancer. Life Sci. Alliance.

[151] Pascual M., Vicente M., Monferrer L., Artero R. (2006). The muscleblind family of proteins: An emerging class of regulators of developmentally programmed alternative splicing. Differentiation.

[152] Park S., Phukan P.D., Zeeb M., Martinez-Yamout M.A., Dyson H.J., Wright P.E. (2017). Structural basis for interaction of the tandem zinc finger domains of human muscleblind with cognate rna from human cardiac troponin t. Biochemistry.

[153] Meola G., Cardani R. (2015). Myotonic dystrophies: An update on clinical aspects, genetic, pathology and molecular pathomechanisms. Biochim Biophys Acta.

[154] Sellier C., Cerro-Herreros E., Blatter M., Freyermuth F., Gaucherot A., Ruffenach F., Sarkar P., Puymirat J., Udd B., Day J.W. (2018). Rbfox1/mbnl1 competition for ccug rna repeats binding contributes to myotonic dystrophy type 1/type 2 differences. Nat. Commun..

[155] Kim E.Y., Barefield D.Y., Vo A.H., Gacita A.M., Schuster E.J., Wyatt E.J., Davis J.L., Dong B., Sun C., Page P. (2019). Distinct pathological signatures in human cellular models of myotonic dystrophy subtypes. Jci. Insight.

[156] Zu T., Cleary J.D., Liu Y., Banez-Coronel M., Bubenik J.L., Ayhan F., Ashizawa T., Xia G., Clark H.B., Yachnis A.T. (2017). Ran translation regulated by muscleblind proteins in myotonic dystrophy type 2. Neuron.

[157] Taneja K.L., McCurrach M., Schalling M., Housman D., Singer R.H. (1995). Foci of trinucleotide repeat transcripts in nuclei of myotonic dystrophy cells and tissues. J. Cell Biol..

[158] Wojciechowska M., Krzyzosiak W.J. (2011). Cellular toxicity of expanded rna repeats: Focus on rna foci. Hum. Mol. Genet..

[159] Van Treeck B., Parker R. (2018). Emerging roles for intermolecular rna-rna interactions in rnp assemblies. Cell.

[160] Smith K.P., Byron M., Johnson C., Xing Y., Lawrence J.B. (2007). Defining early steps in mrna transport: Mutant mrna in myotonic dystrophy type i is blocked at entry into sc-35 domains. J. Cell Biol..

[161] Fardaei M., Larkin K., Brook J.D., Hamshere M.G. (2001). In vivo co-localisation of mbnl protein with dmpk expanded-repeat transcripts. Nucleic Acids Res.

[162] Querido E., Gallardo F., Beaudoin M., Ménard C., Chartrand P. (2011). Stochastic and reversible aggregation of mrna with expanded cug-triplet repeats. J. Cell Sci..

[163] Konieczny P., Stepniak-Konieczna E., Taylor K., Sznajder L.J., Sobczak K. (2017). Autoregulation of mbnl1 function by exon 1 exclusion from mbnl1 transcript. Nucleic Acids Res..

[164] Tran H., Gourrier N., Lemercier-Neuillet C., Dhaenens C.M., Vautrin A., Fernandez-Gomez F.J., Arandel L., Carpentier C., Obriot H., Eddarkaoui S. (2011). Analysis of exonic regions involved in nuclear localization, splicing activity and dimerization of muscleblind-like-1 isoforms. J. Biol. Chem..

[165] Paul S., Dansithong W., Jog S.P., Holt I., Mittal S., Brook J.D., Morris G.E., Comai L., Reddy S. (2011). Expanded cug repeats dysregulate rna splicing by altering the stoichiometry of the muscleblind 1 complex. J. Biol. Chem..

[166] Laurent F.X., Sureau A., Klein A.F., Trouslard F., Gasnier E., Furling D., Marie J. (2012). New function for the rna helicase p68/ddx5 as a modifier of mbnl1 activity on expanded cug repeats. Nucleic Acids Res..

[167] Pettersson O.J., Aagaard L., Andrejeva D., Thomsen R., Jensen T.G., Damgaard C.K. (2014). Ddx6 regulates sequestered nuclear cug-expanded dmpk-mrna in dystrophia myotonica type 1. Nucleic Acids Res..

[168] Ho T.H., Savkur R.S., Poulos M.G., Mancini M.A., Swanson M.S., Cooper T.A. (2005). Colocalization of muscleblind with rna foci is separable from mis-regulation of alternative splicing in myotonic dystrophy. J. Cell Sci..

[169] Xia G., Ashizawa T. (2015). Dynamic changes of nuclear rna foci in proliferating dm1 cells. Histochem Cell Biol..

[170] Dansithong W., Paul S., Comai L., Reddy S. (2005). Mbnl1 is the primary determinant of focus formation and aberrant insulin receptor splicing in dm1. J. Biol. Chem..

[171] Jain A., Vale R.D. (2017). Rna phase transitions in repeat expansion disorders. Nature.

[172] Gudde A.E., González-Barriga A., van den Broek W.J., Wieringa B., Wansink D.G. (2016). A low absolute number of expanded transcripts is involved in myotonic dystrophy type 1 manifestation in muscle. Hum. Mol. Genet..

[173] Wojciechowska M., Sobczak K., Kozlowski P., Sedehizadeh S., Wojtkowiak-Szlachcic A., Czubak K., Markus R., Lusakowska A., Kaminska A., Brook J.D. (2018). Quantitative methods to monitor rna biomarkers in myotonic dystrophy. Sci. Rep..

[174] Zu T., Gibbens B., Doty N.S., Gomes-Pereira M., Huguet A., Stone M.D., Margolis J., Peterson M., Markowski T.W., Ingram M.A. (2011). Non-atg-initiated translation directed by microsatellite expansions. Proc. Natl. Acad. Sci. USA.

[175] Nedelsky N.B., Taylor J.P. (2019). Bridging biophysics and neurology: Aberrant phase transitions in neurodegenerative disease. Nat. Rev. Neurol.

[176] Nguyen L., Cleary J.D., Ranum L.P.W. (2019). Repeat-associated non-atg translation: Molecular mechanisms and contribution to neurological disease. Annu. Rev. Neurosci.

